# Development of a microprocessor-based biochemical sampler

**DOI:** 10.1155/S1463924602000093

**Published:** 2002

**Authors:** S. S. Randhawa, G. Chand, A. K. Ganju

**Affiliations:** Central Scientific Instruments Organisation Chandigarh 160030 India

## Abstract

Modern medicine requires patients to be treated on the basis of precise data, which are often obtained from electronic equipment. An inexpensive and portable microprocessor-based sampler developed by the authors is described. It is comprised of the following units: sample plate assembly, probe-drive linkage system, wash fluid receptacle, timing system and 8085A microprocessor.


Journal of Automated Methods & Management in Chemistry
Vol. 24, No. 2 (March–April 2002) pp. 49–50

### Development of a microprocessor-based biochemical sampler

**S. S. Randhawa*, G. Chand and A. K. Ganju**
*Central Scientific Instruments Organisation, Chandigarh 160030, India*

*Modern medicine requires patients to be treated on the basis of precise data, which are often obtained from electronic equipment. An inexpensive and portable microprocessor-based sampler developed by the authors is described. It is comprised of the following units: sample plate assembly, probe-drive linkage system, wash fluid receptacle, timing system and 8085A microprocessor.*

### Introduction

Automation, or more correctly mechanization, has dominated the clinical biochemistry laboratory's approach to cope with the routine measurement workload. The transition from manual to automated methods in today's laboratories has increased analysis efficiency. The ability to automate samples without losing integrity or data quality is also important. Developments in diagnostic techniques and electronic instruments have made it easier to carry out the various bodily function tests [1–3]. The present paper describes a sampler used in continuous flow analysis for the analysis of biochemical parameters.

### Instrument

A block diagram of the instrument is shown in figure 1. The sample plate is circular in shape and made of anodized aluminium. Twenty-four holes are machined on its circumference, in which sample cups of 5 ml capacity can be placed. A stepper motor drives the plate. The sample probe, which is in the form of a hollow steel tube, is connected to peristaltic pump tubing of the appropriate size, which delivers the sample fluid by the vacuum produced by the pump. With the help of linkages, the probe is made to reciprocate between two fixed positions, i.e. in the sample cup and in the wash receptacle. The wash receptacle is a glass tube provided with water inlet and outlet nipples. They are positioned so that the water level remains constant.

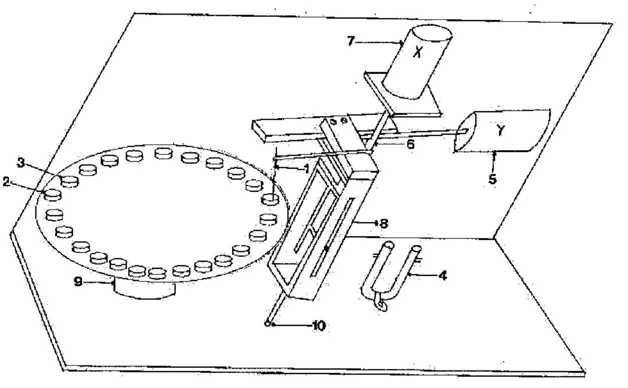

| | | | |
|---|---|---|---|
| 1 | Sample Probe | 7 | Solenoid – X |
| 2 | Sample Tray | 8 | Sample Movement Frame |
| 3 | Sample Cup | 9 | Stepper Motor |
| 4 | Wash Receptacle | 10 | Ball for Rotation |
| 5 | Solenoid – Y | | |
| 6 | Movement Rod | | |

*Figure 1. Block diagram of the sampler.*

In figure 2, the microchip 8085 initalizes chip 8255 through the address line and data line. Since the 8255 chip has three output ports, port C is used as the output port. It can also be used as the reset mode. The output from port C is given to the base of the transistor to drive the relays. The four relays used are designated as A–D. The function of relay A is to operate the solenoid X, which drives the sample probe-linkage system in the upward movement; relay B operates the same solenoid for the downward movement of the sample probe. The function of relay C is to operate the probe in a clockwise direction to 90°. It also operates the stepper motor at an angle of 15° to rotate the sample tray. The function of relay D is to provide the anticlockwise direction through solenoid Y. The operational sequence of the timing system is shown in figure 3.

### Discussion

The sampler is a set of mechanical and electronic controls coupled and synchronized to deliver the sample. The main mechanical system, i.e. the sample plate drive, is powered by a stepper motor and a probe-linkage system operated by solenoids through the relay and controlling circuits (figure 2). It serves as the only inlet point of blood sample/fluid into the reagent train. Since its operation is automatic, the samples are automatically advanced by each step to the aspirating position, where a probe delivers a fixed quantity of sample each time. A timing system incorporated within the electronic controls maintains the desired sample:wash ratio (2:1), which can be selected along with the appropriate relays in the timing system. To maintain a definite gap between samples, the probe sucks in a small quantity of wash solution (distilled water) between each sample. An alarm system gives an

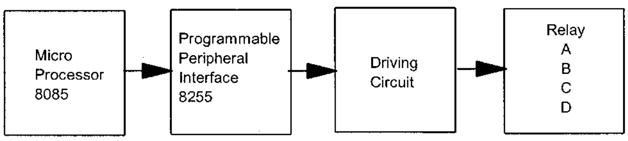

*Figure 2. Block diagram of the electronic circuit of the sampler.*

*To whom correspondence should be addressed.

*Journal of Automated Methods & Management in Chemistry* ISSN 1463–9246 print/ISSN 1464–5068 online © 2002 Taylor & Francis Ltd
http://www.tandf.co.uk/journals
DOI: 10.1080/14639240210121824

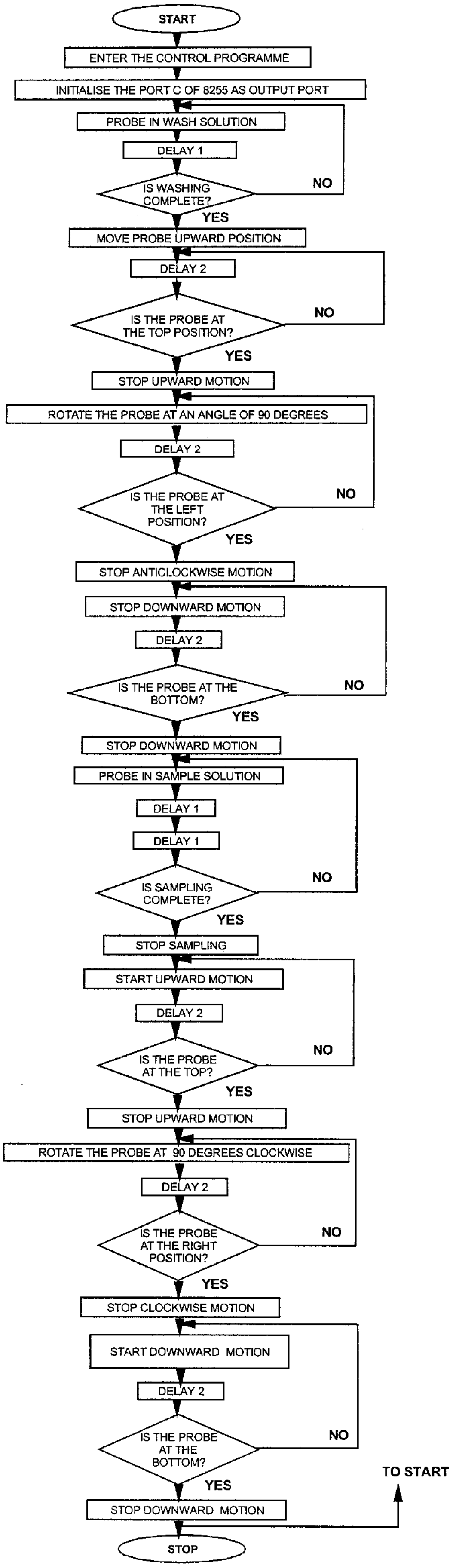

*Figure 3. Operation sequence of the timing system.*

audio signal when a particular batch of samples is completed.

### Conclusion

The sample tray is simple, easy to operate and has a smooth tray movement. It can also be recycled, so that more samples can be analysed.

### Acknowledgement

The authors thank the Director of the Central Scientific Instruments Organisation, Chandigarh, for encouragement, guidance and permission to publish the paper.

### References

1. Rose, A. W. and Steadman, J. W., *Clinical Chemistry*, **20** (1974), 613.
2. Jaffar, M. and Zahid, Q., *Journal of Chemical Education*, **85** (1988), 1099.
3. Randhawa, S. S., Gupta, R. C., Bhjandari, A. K. and Malhotra, P. S., *Journal of Automatic Chemistry*, **14** (1992), 185.

